# Role of PP2Cα in cell growth, in radio- and chemosensitivity, and in tumorigenicity

**DOI:** 10.1186/1476-4598-6-65

**Published:** 2007-10-17

**Authors:** Twan Lammers, Peter Peschke, Volker Ehemann, Jürgen Debus, Boris Slobodin, Sara Lavi, Peter Huber

**Affiliations:** 1 Department of Innovative Cancer Diagnosis and Therapy, Clinical Cooperation Unit Radiotherapeutic Oncology, German Cancer Research Center, Im Neuenheimer Feld 280, 69120 Heidelberg, Germany; 2 Department of Pharmaceutics, Utrecht Institute for Pharmaceutical Sciences (UIPS), Utrecht University, Sorbonnelaan 16, 3508 CA, Utrecht, The Netherlands; 3 Department of Pathology, Heidelberg University Medical School, Im Neuenheimer Feld 220, 69120 Heidelberg, Germany; 4 Department of Radiotherapy and Radiooncology, Heidelberg University Medical School, Im Neuenheimer Feld 400, 69120 Heidelberg, Germany; 5 Department of Cell Research and Immunology, Tel Aviv University, 69978 Tel Aviv, Israel

## Abstract

**Background:**

PP2Cα is the representative member of the type 2C family of protein phosphatases, and it has recently been implicated in the regulation of p53-, TGFβ-, cyclin-dependent kinase- and apoptosis-signaling. To investigate the role of PP2Cα in cell growth and in radio- and chemosensitivity, wild type and PP2Cα siRNA-expressing MCF7 cells were subjected to several different viability and cell cycle analyses, both under basal conditions and upon treatment with radio- and chemotherapy. By comparing the growth of tumors established from both types of cells, we also evaluated the involvement of PP2Cα in tumorigenesis.

**Results:**

It was found that knockdown of PP2Cα did not affect the proliferation, the clonogenic survival and the membrane integrity of MCF7 cells. In addition, it did not alter their radio- and chemosensitivity. For PP2Cα siRNA-expressing MCF7 cells, the number of cells in the G0/G1 phase of the cell cycle was reduced, the induction of the G1 block was attenuated, the number of cells in G2/M was increased, and the induction of the G2 block was enhanced. The tumorigenic potential of PP2Cα siRNA-expressing MCF7 cells was found to be higher than that of wild type MCF7 cells, and the in vivo proliferation of these cells was found to be increased.

**Conclusion:**

Based on these findings, we conclude that PP2Cα is not involved in controlling cell growth and radio- and chemosensitivity in vitro. It does, however, play a role in the regulation of the cell cycle, in the induction of cell cycle checkpoints and in tumorigenesis. The latter notion implies that PP2Cα may possess tumor-suppressing properties, and it thereby sets the stage for more elaborate analyses on its involvement in the development and  progression of cancer.

## Background

Over the past few decades, significant progress has been made in understanding the principles of malignant transformation and tumorigenesis. The overexpression and/or overactivation of protein kinases, for instance, like EGFR, Her2/neu, Bcr-Abl, c-Met and c-Kit, has been repetitively shown to be causally linked to the development of malignancy, and it has resulted in the establishment of several new classes of anticancer agents, like receptor-binding antibodies and tyrosine kinase inhibitors. The roles and the functions of the physiological inhibitors of protein kinases, however, i.e. of protein phosphatases, have been far less well-documented, and only for a short while now, significant effort has been put in evaluating the involvement of these enzymes in cancer development and/or tumor suppression.

The type 2C family of protein phosphatases is a structurally and functionally distinct group of enzymes that currently contains about 15 different family members [1,2]. As opposed to other serine-threonine specific phosphatases, PP2Cs are known to function as monomers, to depend on bivalent cations for their activity and to be insensitive to the broad-spectrum phosphatase-inhibitor okadaic acid [1-3]. Except for the well-known oncoprotein PP2Cδ (PPM1D/Wip1) [4-6], all PP2Cs to which functions have been attributed have been shown to act as inhibitors of cell growth and cellular stress signaling [2,7-9].

PP2Cα, for instance, the representative member of the type 2C family of protein phosphatases, upregulates the expression and the activity of p53 [10,11], it reduces the activities of the growth-promoting cyclin-dependent kinases Cdk2 and Cdk6 [12,13], it negatively affects TGFβ-signaling [14], it is involved in apoptosis induction [15,16], and it inhibits the two stress-activated MAPK pathways JNK and p38 [17,18]. PP2Cβ has also been shown to activate p53 [11], to inactivate Cdk2 and Cdk6 [12,13], and to inhibit JNK and p38 [17,19]. In addition, it has been shown to dephosphorylate and activate the proapoptotic protein BAD, thereby enabling BAD to neutralize the antiapoptotic effects of Bcl-xL [20]. And furthermore, by dephosphorylating and inactivating IKKβ, PP2Cβ has also been implicated in the attenuation of NF-kB-mediated antiapoptosis [21]. The recently identified type 2C phosphatase ILKAP (integrin-linked kinase-associated phosphatase) is yet another example of a PP2C family member with potent growth-suppressive properties [22]. By dephosphorylating and inactivating the integrin-linked kinase ILK1, which is involved in the activation of the oncogenic ILK1-GSK3β-Wnt pathway and which is known to be overexpressed in several different human malignancies [23-25], ILKAP has been shown to be able to inhibit both the proliferation and the malignant transformation of cells [26].

Based on the abovementioned observations, and on the fact that several others PP2Cs (e.g. PP2Cγ, PP2Cε, PHLPP, CaMKP and CaMKP-N) have also been implicated in the inhibition of cell growth and cellular stress signaling [27-30], we reasoned that type 2C phosphatases may play a general physiological role in the regulation of these two processes. To assess the validity of this assumption, we investigated the involvement of the prototypic type 2C phosphatase PP2Cα in cell growth, in radio- and chemosensitivity, and in tumorigenicity. Hereto, the proliferation, the clonogenic survival, the membrane integrity and the cell cycle distribution of wild type and PP2Cα siRNA-expressing MCF7 cells were analyzed, both under basal conditions and upon treatment with different doses of radio- and chemotherapy. The role of PP2Cα in tumorigenesis was evaluated by comparing the growth, the cell cycle distribution and the proliferation rate of tumors established from both types of cells. It was found that the knockdown of PP2Cα affected the in vitro growth and the radio- and chemosensitivity of MCF7 cells only very moderately. It did, however, reflect on their cell cycle distribution and their tumorigenic potential. In line with the notions that PP2Cα activates p53 [10,11] and inactivates Cdk2 and Cdk6 [12,13], these findings suggest that PP2Cα is involved in cell cycle regulation and in tumor suppression.

## Results

### Characterization of PP2Cα-siRNA expressing MCF7 cells

**Figure 1:**
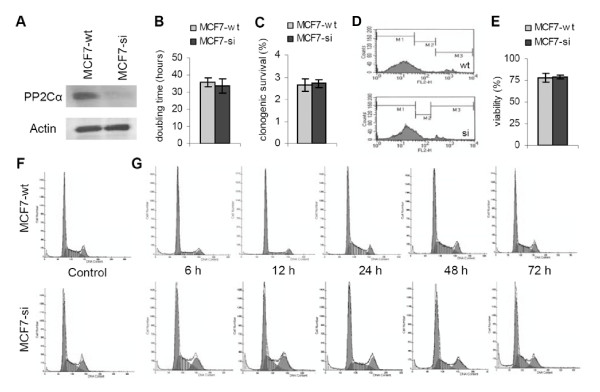
**Characterization of wild type and PP2Cα siRNA-expressing MCF7 cells**. A: Western blot analysis of the expression of PP2Cα and Actin in wild type (MCF7-wt) and PP2Cα knockdown (MCF7-si) cells. B: Doubling time of MCF7-wt and MCF7-si cells. Values represent average ± SD (n = 3). C: Clonogenicity of MCF7-wt and MCF7-si cells. Values represent average ± SD (n = 5). D: Representative FACS analyses of the uptake of propidium iodide (PI) by confluently growing MCF7-wt and MCF7-si cells. The uptake of PI was used to determine the membrane integrity of the (unfixed) cells. M1: viable cells. M2: membrane-damaged cells. M3: dead cells. E: Quantification of the viability of MCF7-wt and MCF7-si cells. The amount of viable cells (M1 fraction) was determined by pooling the data obtained in three independent analyses. Values represent average ± SD. F: Representative images of the cell cycle distribution of confluently growing MCF7-wt and MCF7-si cells. G: Representative images of the cell cycle distribution of MCF7-wt and MCF7-si cells at various time points after trypsination and (re-) seeding.

PP2Cα siRNA-expressing MCF7 cells (MCF7-si) were prepared using the pSUPER-Retro vector [31]. The western blot analyses in Fig. 1A demonstrate that as expected, as compared to wild type MCF7 cells (MCF7-wt), MCF7-si cells expressed substantially reduced amounts of PP2Cα. To investigate the impact of PP2Cα knockdown on cell growth in vitro, we started off by analyzing the doubling times of MCF7-wt and MCF7-si cells. As shown in Fig. 1B, no significant difference was observed between the two types of cells; 35.6 ± 2.7 h was found for MCF7-wt, as compared to 33.7 ± 4.3 h for MCF7-si (p = 0.55). Next, the effect of reducing the expression of PP2Cα on the clonogenicity of the cells was investigated. Fig. 1C demonstrates that also in this case, no significant difference could be observed between MCF7-wt and MCF7-si cells: 2.66 ± 0.28% and 2.72 ± 0.19% of the initial amount of seeded cells were able to form colonies, respectively (p = 0.70). Subsequently, the membrane integrity of MCF7-wt and MCF7-si cells was compared. This was done by trypsinizing and harvesting confluently growing cells, and by analyzing the uptake of propidium iodide (PI: a membrane-impermeable dye that only enters and stains membrane-damaged and dead cells) by these unfixed cells. As shown in the plots in Fig. 1D, three fractions of cells can be generally discriminated, i.e. viable cells (low PI uptake; M1-fraction), membrane-damaged cells (intermediate PI uptake; M2-fraction) and dead cells (high PI uptake; M3-fraction). Fig. 1D also shows that there was hardly any difference in membrane integrity between MCF7-wt and MCF7-si cells. This notion was confirmed by averaging the data obtained in three independent analyses: of the MCF-wt cells, 78.2 ± 5.3% were viable, 13.2 ± 3.5% were membrane-damaged and 8.9 ± 2.0% were dead, and of the MCF-si cells, 79.3 ± 2.3% were viable, 13.4 ± 2.4% were membrane-damaged and 7.6 ± 1.5% were dead (Fig. 1E; p > 0.05 for all comparisons). These findings indicate that the knockdown of PP2Cα does not alter the 'baseline-viability' of MCF7 cells.

### Effect of PP2Cα knockdown on cell cycle distribution

Next, confluently growing MCF7-wt and MCF7-si cells were trypsinized, harvested, fixed, labeled with PI, and subjected to cell cycle analysis. Fig. 1F shows that the knockdown of PP2Cα resulted in an increase in the number of cells in G2/M. This observation was confirmed by pooling the data obtained in three independent analyses; 22.7 ± 3.7% of the MCF7-si cells were in G2/M, as compared to 12.0 ± 1.2% for MCF7-wt cells (p = 0.009; Table 1). Table 1 also demonstrates that the knockdown of PP2Cα resulted in a reduction of the number of cells in G0/G1; 44.9 ± 2.4% of the MCF7-si cells were in G0/G1, as compared to 54.5 ± 1.5% for MCF7-wt (p = 0.004). The percentages of cells in the S phase were comparable (Table 1; p = 0.30).

**Table 1:** Cell cycle analysis of wild type and PP2Cα siRNA-expressing MCF7 cells.

**Time (hours post trypsination)**	**MCF7-wt**			**MCF7-si**		
	**G0/G1**	**S**	**G2/M**	**G0/G1**	**S**	**G2/M**
Control	54.5 ± 1.5	33.5 ± 0.4	12.0 ± 1.3	44.9 ± 2.4 *	32.4 ± 1.6	22.7 ± 3.7 *
6	74.0 ± 0.5	17.3 ± 0.2	8.6 ± 0.4	50.2 ± 2.8 *	34.4 ± 2.4 *	16.1 ± 4.2 *
12	74.1 ± 0.2	16.7 ± 0.8	9.2 ± 0.7	46.1 ± 3.6 *	33.5 ± 3.1 *	20.4 ± 6.7 *
24	48.0 ± 0.9	41.7 ± 0.4	10.3 ± 0.9	57.8 ± 2.7 *	23.8 ± 3.5 *	18.4 ± 1.1 *
48	48.4 ± 0.7	39.9 ± 0.4	11.6 ± 0.8	59.4 ± 4.8 *	25.1 ± 0.3 *	15.5 ± 4.9
72	51.9 ± 0.5	34.6 ± 0.5	13.5 ± 0.3	46.3 ± 2.7 *	31.3 ± 1.0 *	21.8 ± 2.5 *

Multicycle algorithm-based quantification of the amounts of MCF7-wt and MCF7-si cells in the G0/G1, the S and the G2/M phase of the cell cycle. Results are shown both for confluently growing control cells and for cells analyzed at various time points after trypsination and (re-) seeding. Values represent average ± SD (n = 3). * Indicates p < 0.05 vs. MCF-wt.

Subsequently, the cell cycle distribution of the two types of cells was investigated at various time points after trypsinization and (re-) seeding. Fig. 1G and Table 1 show that also upon trypsinization, MCF7-si cells presented with increased amounts in G2/M and with decreased percentages in G0/G1. The fact that the number of MCF7-si cells in G2/M was found to be higher at all time points post reseeding (p < 0.05 for all, except for 48 h; Table 1) indicates that PP2Cα can be expected to play a general physiological role in the regulation of the G2-to-M transition. The percentages in G0/G1 showed a different pattern; at the early (6 and 12 h) and late (72 h) time points post reseeding, the number of cells in G0/G1 was significantly lower for MCF7-si. At 24 and 48 h, however, their percentages in G0/G1 were significantly higher. This suggests that in cells lacking PP2Cα, the induction of the G0/G1 arrest in response to trypsinization is delayed (peak at 24–48 h for MCF7-si vs. at 6–12 h for MCF7-wt). Together with the notion that the maximal extent of the G0/G1 arrest was lower for MCF7-si cells (59% vs. 74%; Table 1), this observation indicates that PP2Cα likely also plays a role in the induction the G1 block and in the G1-to-S transition.

### Effect of PP2Cα knockdown on radiosensitivity

**Figure 2:**
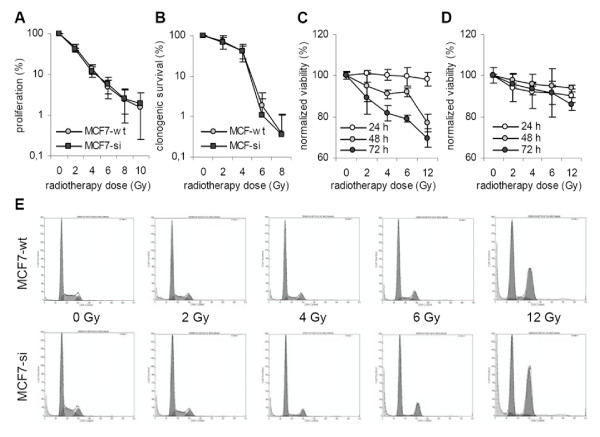
**Evaluation of the radiosensitivity of wild type and PP2Cα siRNA-expressing MCF7 cells**. A: Effect of different doses of ^60^Cobalt γ-radiation on the proliferation of MCF7-wt and MCF7-si cells. Values represent average ± SD (n = 4). B: Effect of radiotherapy on the clonogenicity of MCF7-wt and MCF7-si cells. Values represent average ± SD (n = 4). C and D: Quantification of the effect of radiotherapy on the viability (i.e. the membrane integrity) of MCF7-wt and MCF7-si cells. The viability was determined by means of FACS analysis. Values represent average ± SD (n = 3). E: Representative images of the cell cycle distribution of MCF7-wt and MCF7-si cells at 48 h after treatment with radiotherapy.

The role of PP2Cα in regulating radiosensitivity was investigated by exposing wild type and siRNA-expressing MCF7 cells to different doses of ^60^Cobalt γ-radiation, and by subsequently subjecting them to three different viability assays. The Sulphorhodamine B (SRB) assay was used to assess cellular proliferation [32]. Fig. 2A shows that there was no significant difference in proliferation between MCF7-wt and MCF7-si cells in response to radiotherapy. Fig. 2B shows that also when analyzing the clonogenic survival of the cells, no difference could be observed between the two types of cells. Analysis of the membrane integrity of the cells in response to radiotherapy, on the other hand, did point towards a difference between wild type and PP2Cα knockdown cells; as shown in Fig. 2C, as expected, the percentages of physiologically intact MCF7-wt cells decreased as I) the time post-treatment increased and as II) the dose of radiotherapy increased; 48 h after 6 and 12 Gy, for instance, the normalized viabilities were 92.0 ± 2.2% and 77.0 ± 4.4%, respectively. For MCF7-si cells, on the other hand, the degree of membrane-damage did not correlate to the time post treatment and to the dose of radiotherapy applied; 48 h after 6 and 12 Gy, the normalized viabilities were 94.9 ± 0.7% (p = 0.30) and 93.8 ± 2.6% (p = 0.004), respectively. These findings indicate that the knockdown of PP2Cα does not alter the proliferation and the clonogenic survival of MCF7 cells in response to radiotherapy, but that it does reflect on their membrane integrity.

The impact of PP2Cα knockdown on radiotherapy-induced cell cycle changes was investigated by exposing the MCF7-wt and the MCF7-si cells to 0, 2, 4, 6, and 12 Gy of ^60^Cobalt γ-radiation, and by analyzing their cell cycle distribution 24, 48 and 72 h later. Fig. 2E shows representative results obtained at 48 h after radiotherapy. As can be seen, the only two differences between the two types of cells were that I) the number of sham-irradiated control cells in the G2/M phase again tended to be higher for MCF7-si (in line the data obtained under basal conditions (Fig. 1F and Table 1)), and that II) the induction of the G2 block in response to high doses of radiotherapy was stronger for MCF7-si.

Quantitative analysis using the Multicycle algorithm confirmed that under control conditions (i.e. at 24 h after exposure to 0 Gy), the percentage in G2/M was significantly higher for PP2Cα knockdown cells (14.2 ± 0.7% for MCF7-si, as compared to 9.2 ± 1.5% (p = 0.007) for MCF7-wt; Table 2). It also again showed that this increase in G2/M was paralleled by a decrease in G0/G1; 55.6 ± 0.5% were found in the G0/G1 phase for MCF-si, as compared to 61.3 ± 0.5% for MCF-wt (p = 0.0001). These notions strengthen the conclusion that PP2Cα is involved in cell cycle regulation.

**Table 2:** Cell cycle analysis of wild type and PP2Cα siRNA-expressing MCF7 cells upon treatment with radiotherapy.

		**MCF7-wt**			**MCF7-si**		
**Time (h)**	**RT Dose (Gy)**	**G0/G1**	**S**	**G2/M**	**G0/G1**	**S**	**G2/M**
24	0	61.3 ± 0.5	29.5 ± 1.1	9.2 ± 1.5	55.6 ± 0.5 *	30.3 ± 0.4	14.2 ± 0.7 *
	2	68.3 ± 0.2	21.8 ± 0.4	9.9 ± 0.4	61.4 ± 1.3 *	25.4 ± 1.1 *	13.2 ± 0.5 *
	4	71.6 ± 0.2	16.7 ± 0.2	11.7 ± 0.3	67.4 ± 6.2 *	25.0 ± 5.7 *	7.5 ± 0.9 *
	6	70.9 ± 0.6	14.0 ± 0.4	15.1 ± 0.7	77.5 ± 0.9 *	9.2 ± 1.5 *	13.3 ± 0.6 *
	12	66.8 ± 0.5	12.6 ± 0.3	20.6 ± 0.5	54.8 ± 0.9 *	3.4 ± 0.3 *	41.8 ± 0.8 *

48	0	61.5 ± 0.6	35.4 ± 1.4	12.7 ± 1.3	61.2 ± 1.8 *	31.0 ± 1.2 *	12.0 ± 0.4
	2	62.9 ± 8.0	28.1 ± 5.5	9.0 ± 2.5	69.2 ± 0.4	21.4 ± 0.2	9.4 ± 0.4
	4	75.9 ± 6.7	15.8 ± 8.0	8.2 ± 1.3	77.9 ± 0.2	11.3 ± 0.4	10.8 ± 0.6 *
	6	79.2 ± 1.0	7.4 ± 2.8	13.7 ± 3.4	74.4 ± 0.9 *	5.8 ± 0.4	19.8 ± 1.3 *
	12	55.3 ± 1.1	7.2 ± 3.7	37.3 ± 3.1	48.8 ± 0.4 *	2.6 ± 0.4	48.6 ± 0.4 *

72	0	51.1 ± 1.3	42.4 ± 2.0	6.4 ± 1.2	54.0 ± 2.6	33.6 ± 0.2 *	12.6 ± 2.9 *
	2	61.1 ± 0.3	26.6 ± 1.1	12.3 ± 1.0	61.9 ± 0.6	24.7 ± 0.1 *	13.4 ± 0.6
	4	65.1 ± 1.7	21.2 ± 1.1	13.7 ± 1.3	66.2 ± 0.9	18.5 ± 0.9 *	15.3 ± 1.5
	6	66.4 ± 0.6	14.7 ± 0.8	18.9 ± 0.9	68.4 ± 0.6 *	13.8 ± 0.4	17.9 ± 0.1
	12	51.7 ± 0.7	8.1 ± 0.6	40.3 ± 0.2	54.9 ± 0.3 *	6.4 ± 0.8	38.7 ± 0.2 *

Multicycle algorithm-based quantification of the amounts of MCF7-wt and MCF7-si cells in the G0/G1, the S and the G2/M phase of the cell cycle at 24, 48 and 72 h after treatment with the indicated doses of radiotherapy. Values represent average ± SD (n = 3). * Indicates p < 0.05 vs. MCF-wt.

Overall, the observed radiotherapy-induced cell cycle changes were in accordance with the literature (33-35), as cells generally (attempt to) repair radiation-induced DNA damage in the two gap phases of the cycle, i.e. in G1 and G2; at lower doses of radiotherapy, both MCF7-wt and MCF7-si cells presented with increases in G0/G1, and at higher doses, both presented with increases in G2/M (Table 2). When focusing on the latter, two obvious differences could be noted between the two types of cells. First, a strong induction of the G2 block presented earlier on in time in the MCF7-si cells; at 24 h, MCF7-si cells already presented with a strong arrest in G2/M upon exposure to 12 Gy (41.8 ± 0.8%), whereas MCF7-wt cells only displayed a slight induction of the G2 block at this time point (20.6 ± 0.5%; p < 0.0001). And second, the overall extent of the G2 block was higher for MCF7-si; 48 h after 12 Gy, for instance, 48.6 ± 0.4% was found in G2/M for MCF7-si, as compared to 37.3 ± 3.1% for MCF7-wt (p = 0.003; Table 2). These observations indicate that the knockdown of PP2Cα affects the radiotherapy-induced changes in the cell cycle distribution of MCF7 cells both in a time-dependent manner (earlier induction of the G2 block) and in a concentration-dependent manner (stronger induction of the G2 block).

### Effect of PP2Cα knockdown on chemosensitivity

**Figure 3:**
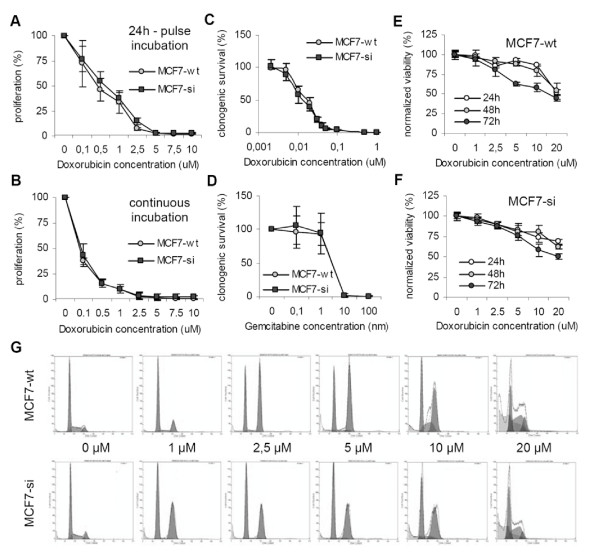
**Evaluation of the chemosensitivity of wild type and PP2Cα siRNA-expressing MCF7 cells**. A and B: Effect of different doses of doxorubicin on the proliferation of MCF7-wt and MCF7-si cells. The cells were either exposed to the drug for 24 h (A) or continuously (B). Values represent average ± SD (n = 3). C: Effect of doxorubicin on the clonogenicity of MCF7-wt and MCF7-si cells. Values represent average ± SD (n = 3). D: Effect of gemcitabine on the clonogenicity of MCF7-wt and MCF7-si cells. Values represent average ± SD (n = 3). E and F: Quantification of the effect of doxorubicin on the viability (i.e. the membrane integrity) of MCF7-wt and MCF7-si cells. The viability was determined by means of FACS analysis. Values represent average ± SD (n = 3). G: Representative images of the cell cycle distribution of MCF7-wt and MCF7-si cells after 48 h of doxorubicin treatment.

To investigate the impact of PP2Cα knockdown on chemosensitivity, wild type and siRNA-expressing MCF7 cells were exposed to doxorubicin and subjected to the three abovementioned viability assays. Figs. 3A and 3B demonstrate that at doxorubicin concentrations of 0.1–10 μM, no difference in proliferation could be observed between the two types of cells, neither when they were pulse-incubated (i.e. for 24 h; Fig. 3A), nor when they were incubated continuously (i.e. for 120 h; Fig. 3B). Next, the impact of doxorubicin on the clonogenic survival of wild type and PP2Cα knockdown cells was evaluated. As shown in Fig. 3C, also in this case, no difference in response to chemotherapeutic treatment could be observed between the two types of cells. To exclude the possibility that the observed lack of an altered chemosensitivity was drug-specific, we also evaluated the clonogenicity of the cells in response to gemcitabine. As shown in Fig. 3D, however, also upon treatment with gemcitabine, no difference in clonogenic survival could be detected between wild type and PP2Cα knockdown cells. This finding indicates that the observed lack of a differential chemosensitivity was not specific for doxorubicin, and it suggests that MCF7-wt and MCF-si cells are equally sensitive towards chemotherapeutic treatment. Subsequently, as for radiotherapy, we then also investigated the impact of chemotherapy on the membrane integrity of MCF7-wt and MCF7-si cells. Figs. 3E and 3F present the pooled results of three independent analyses, and they demonstrate that there was hardly any difference in chemosensitivity between the two types of cells; at 24 h, the uptake of PI was slightly higher for MCF7-si, at 48 h, the viability of the cells was identical, and at 72 h, the degree of membrane-damage was slightly higher for MCF7-wt. In line with information obtained in the SRB and the CFA analyses, these findings demonstrate that the knockdown of PP2Cα does not alter the chemosensitivity of MCF7 cells.

To assess the effects of the knockdown of PP2Cα on the chemotherapy-induced cell cycle changes, MCF7-wt and MCF7-si cells were incubated with doxorubicin concentrations ranging from 1 to 20 μM and subjected to cell cycle analysis 24, 48 and 72 h later. As shown in Fig. 3G and Table 3, three more or less obvious differences could be observed between the two cell types: I) whereas the former presented with increases in the number of cells in both G0/G1 and G2/M at 24 and 48 h after incubation with 1 μM of doxorubicin, the latter presented with decreases in G0/G1 and, at 48 h, with a much more substantial increase in G2/M. II) In the MCF7-si cells, a strong induction of the G2 block upon exposure to 1 μM of doxorubicin presented earlier on time (at 48 h) than in the MCF7-wt cells (at 72 h). III) In the MCF7-si cells, the maximal induction of the G2 block was lower than that in MCF7-wt cells (57.8 ± 1.5% vs. 63.2 ± 1.1%). These results demonstrate that without affecting the proliferation, the clonogenic survival and the membrane integrity of MCF7 cells, the knockdown of PP2Cα does reflect, at least to some extent, on their cell cycle distribution.

**Table 3:** Cell cycle analysis of wild type and PP2Cα siRNA-expressing MCF7 cells upon treatment with chemotherapy.

		**MCF7-wt**			**MCF7-si**		
**Time (h)**	**Dox Dose (μM)**	**G0/G1**	**S**	**G2/M**	**G0/G1**	**S**	**G2/M**
24	0	53.6 ± 0.5	33.3 ± 1.2	13.1 ± 0.3	56.6 ± 0.5 *	29.7 ± 0.6 *	13.7 ± 0.9
	1	74.8 ± 0.9	8.0 ± 0.6	17.2 ± 0.3	53.9 ± 0.6 *	30.7 ± 1.0 *	15.4 ± 1.3
	2.5	50.4 ± 0.7	4.1 ± 0.8	45.5 ± 1.5	52.1 ± 0.9	5.0 ± 2.7	42.8 ± 1.9
	5	45.1 ± 0.2	6.4 ± 0.2	48.5 ± 0.1	51.5 ± 1.6 *	5.1 ± 2.4	43.4 ± 0.9 *
	10	38.9 ± 1.3	29.5 ± 0.8	31.6 ± 0.8	41.3 ± 0.3 *	32.5 ± 1.6 *	26.2 ± 1.5 *
	20	40.8 ± 1.8	31.3 ± 2.5	27.8 ± 1.3	54.9 ± 1.4 *	30.2 ± 0.5	14.9 ± 1.4 *

48	0	61.5 ± 0.6	29.1 ± 1.0	9.4 ± 0.7	61.2 ± 1.8	29.5 ± 1.7	11.2 ± 0.1 *
	1	71.4 ± 3.5	8.9 ± 1.7	19.7 ± 5.2	52.2 ± 2.7 *	2.5 ± 1.2 *	45.3 ± 1.7 *
	2.5	35.7 ± 1.3	3.9 ± 0.3	60.4 ± 1.0	58.3 ± 5.8 *	3.2 ± 0.6	38.5 ± 5.3 *
	5	31.7 ± 0.5	5.2 ± 1.6	63.2 ± 1.1	49.6 ± 1.0 *	2.5 ± 0.7	47.8 ± 0.4 *
	10	30.3 ± 1.0	23.6 ± 0.8	46.1 ± 0.7	44.0 ± 1.7 *	8.4 ± 0.6 *	47.6 ± 2.3
	20	33.2 ± 1.8	48.6 ± 7.7	18.3± 9.4	50.3 ± 0.8 *	43.4 ± 2.4	6.4 ± 1.6

72	0	71.9 ± 1.7	22.8 ± 0.7	5.4 ± 1.3	59.2 ± 0.9 *	30.4 ± 1.6 *	10.5 ± 0.6 *
	1	52.5 ± 7.2	3.1 ± 1.9	44.4 ± 8.9	47.4 ± 7.3	0.5 ± 0.9	52.1 ± 8.2
	2.5	46.4 ± 1.2	0.5 ± 0.5	53.3 ± 2.0	41.3 ± 2.6 *	1.9 ± 3.0	56.8 ± 1.8
	5	38.8 ± 0.3	0.1 ± 0.1	61.3 ± 0.3	40.4 ± 0.8 *	1.8 ± 1.6	57.8 ± 1.5 *
	10	34.1 ± 0.7	16.4 ± 1.7	49.5 ± 2.3	37.5 ± 1.5 *	3.5 ± 2.5 *	57.1 ± 2.6 *
	20	32.8 ± 2.8	39.9 ± 1.8	27.3 ± 1.8	N.Q.	N.Q.	N.Q.

Multicycle algorithm-based quantification of the amounts of MCF7-wt and MCF7-si cells in the G0/G1, the S and the G2/M phase of the cell cycle at 24, 48 and 72 h after treatment with the indicated doses of doxorubicin. Values represent average ± SD (n = 3). N.Q. (Not Quantifiable) indicates that multicycle algorithm failed to attribute the cells to a certain cell cycle phase. * Indicates p < 0.05 vs. MCF-wt.

### Tumorigenicity of wild type and PP2Cα knockdown MCF7 cells

**Figure 4:**
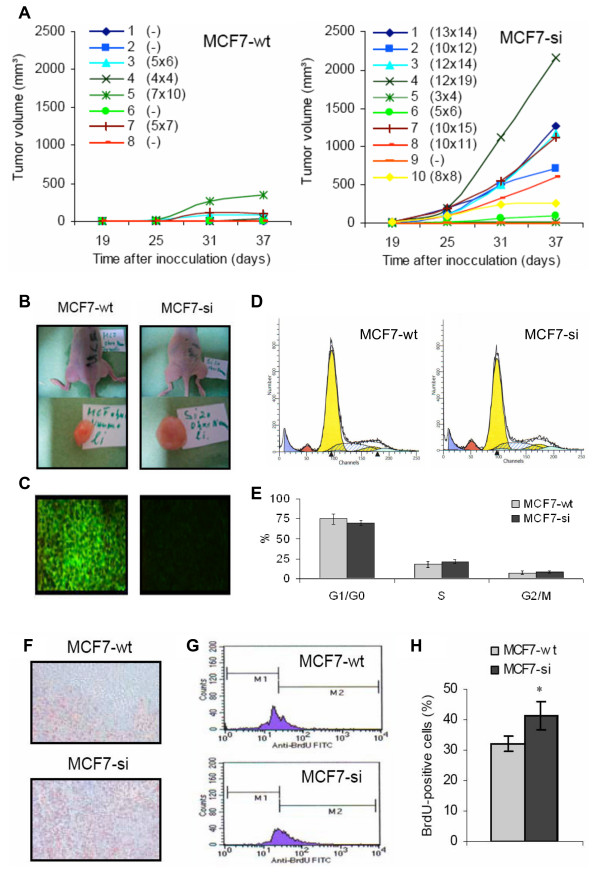
**Evaluation of the tumorigenic potential of wild type and PP2Cα siRNA-expressing MCF7 cells**. A: Tumor growth curves obtained for subcutaneously inoculated MCF7-wt and MCF7-si cells upon the combined implementation of estrogen-containing hormone pellets and Matrigel. The values indicate the individual tumor sizes at day 37. B: Exemplary images of MCF7-wt and MCF7-si tumors at day 37 post inoculation. C: Analysis of the expression of PP2Cα in MCF7-wt and MCF7-si tumors. Magnification 200×. D: Representative images of the cell cycle distribution of MCF7-wt and MCF7-si tumors. Left yellow peak: G0/G1 fraction of the aneuploid tumor cells (i.e. the MCF7 cells). Right yellow peak: G2/M fraction of the aneuploid tumor cells. Dashed blue area: S phase fraction of the aneuploid tumor cells. Red peak: G1 fraction of the diploid host cells (e.g. mouse fibroblasts and endothelial cells). Blue peak: Necrotic cells. E: Multicycle algorithm-based quantification of the cell cycle distribution of MCF7-wt and MCF7-si tumors. Values represent average ± SD (n = 3). F: Analysis of the expression of the proliferation marker Ki-67 in MCF7-wt and MCF7-si tumors. Magnification 200×. G: Representative FACS analyses of the incorporation of the proliferation marker BrdU in MCF7-wt and MCF7-si tumors. H: Quantification of the amount of BrdU-positive cells in MCF7-wt and MCF7-si tumors. Values represent average ± SD (n = 3). * Indicates p < 0.05.

To address the involvement of PP2Cα in tumorigenesis, 1 * 10^7 ^MCF-wt and MCF7-si cells were inoculated subcutaneously (s.c.) into both hind limbs of immunodeficient nude mice. Neither the MCF7-wt cells, however, nor the MCF7-si cells turned out to be able to develop tumors under physiological conditions (Table 4). Based on the notion that MCF7 cells are hormone-dependent breast cancer cells, and on the assumption that their in vivo growth therefore likely depends on the levels of circulating hormones, we next implanted estrogen-containing hormone pellets into 8 nude mice. Ten days after the implantation of the pellets, 1 * 10^7 ^MCF7-wt and MCF7-si cells were again inoculated s.c. into both hind limbs of the animals. As shown in Table 4, in one animal injected with MCF7-si cells, a tumor had formed (8 × 11 mm at day 35). In the other three animals injected with MCF7-si cells, and in all four animals injected with MCF7-wt cells, no tumors had developed. In order to increase the number of malignant cells remaining at the site of inoculation, we subsequently suspended 1 * 10^7 ^cells in Matrigel and inoculated them s.c. into 10 nude mice. Up to three weeks post inoculation, 2–3 mm sized tumors (or rather, semi-palpable suspensions of tumor cells) could be observed in all animals. After this time point, however, they disappeared one after the other, and at day 50, no tumors had developed (Table 4). Finally, in an ultimate attempt to improve the tumorigenic potential of the two types of MCF7 cells, the estrogen-containing hormone pellets were combined with Matrigel; hereto, 1 * 10^7 ^wild type and PP2Cα knockdown cells were suspended in Matrigel, and they were inoculated into mice that had been implanted with pellets 10 days before. Approximately 3 weeks after inoculation, several animals, especially those injected with MCF7-si cells, started to present with signs of tumor growth. As shown in Figs. 4A and 4B, from this day on, several MCF7-si tumors kept on growing steadily, reaching sizes of e.g. 13 × 14 or 10 × 12 mm by day 37. The growth curves in Fig. 4A furthermore demonstrate that a substantially smaller fraction of the animals inoculated with MCF7-wt cells turned out to be able to form tumors (4 out of 8, as compared to 9 out of 10 for MCF7-si), and they also exemplify that those MCF7-wt tumors that did grow, grew much slower than did the MCF7-si tumors; 37 days after inoculation, the largest MCF7-wt tumor was 7 × 10 mm, as compared to 12 × 19 mm for MCF7-si (Figs. 4A and 4B). These findings suggest that, at least under certain conditions, the knockdown of PP2Cα increases the tumorigenicity of MCF7 cells.

**Table 4:** Overview over the experiments evaluating the tumorigenicity of the MCF7-wt and MCF7-si cells.

**Experiment**	**Hormone Pellets**	**Matrigel**	**MCF-wt**	**MCF-si**
**1**	-	-	0/10	0/8
**2**	+	-	0/8	1/8
**3**	-	+	0/10	0/10
**4**	+	+	4/8	9/10

The number of tumors that had developed under control conditions (Exp. 1), upon the implantation of estrogen-containing hormone pellets (Exp. 2), upon the use of Matrigel (Exp. 3), and upon the combined implementation of estrogen-containing hormone pellets and Matrigel (Exp. 4) are presented.

To validate that the MCF7-wt and MCF7-si tumors that had developed upon the combined implementation of the hormone pellets and Matrigel presented with physiological and attenuated amounts of PP2Cα, respectively, the expression of PP2Cα was analyzed immunohistochemically. As shown in the images in Fig. 4D, in MCF7-wt tumors, substantial amounts of PP2Cα could be detected. In MCF7-si tumors, on the other hand, hardly any expression of PP2Cα could be observed. This finding indicates that even after having grown in vivo for more than five weeks, the siRNA-producing pSUPER-Retro vector was still fully functional.

To evaluate how the knockdown of PP2Cα reflects on the in vivo cell cycle distribution of the MCF7 cells, PFA-fixed tumor material was subjected to cell cycle analysis. As shown in Figs. 4D and 4E, in line with the in vitro studies, the number of MCF7-si cells in G0/G1 tended to be decreased and the amounts in G2/M tended to be increased. In addition, the percentages in S appeared to be increased. Quantification using the Multicycle algorithm, however, demonstrated that neither of the three differences was statistically significant (Fig. 4E).

To investigate the impact of PP2Cα knockdown on cell growth in vivo, the expression of the proliferation marker Ki-67 was analyzed in MCF7-wt and MCF7-si tumors. As shown in Fig. 4F, no difference in the intensity of Ki-67 staining could be observed. The pattern of Ki-67 staining, however, was repetitively found be different, with MCF7-si tumors being stained more homogenously than MCF7-wt tumors; for the former, virtually all areas investigated displayed expression of the proliferation marker, whereas for the latter, only certain areas clearly displayed positive signals.

To quantitatively confirm this notion, we finally also assessed cell growth in vivo by means of BrdU staining. BrdU is a synthetic thymidine analogue that is incorporated into DNA during replication and that is routinely used as a marker for cells in the S phase, i.e. for proliferating cells. As shown in the exemplary FACS analyses in Fig. 4G, the incorporation of BrdU tended to be higher for PP2Cα knockdown tumors. Quantitative analyses confirmed that the extent of BrdU incorporation was significantly higher for MCF7-si (41.3 ± 4.7%, vs. 31.9 ± 2.4% for MCF7-wt; p = 0.036; Fig. 4H). Together with the notion that the staining of Ki-67 was more homogenous for MCF7-si, this finding indicates that in PP2Cα knockdown tumors, a higher proportion of the cells contributes to the overall growth of the tumors, and it explains, at least in part, the more effective and the more rapid growth of these tumors.

## Discussion

Ever more interesting features, phenotypes and potential clinical applications are being ascribed to the type 2C family of protein phosphatases. PP2Cα, for instance, has been shown to activate p53 [10], to inhibit MDM2 [11], to inactivate Cdk2 and Cdk6 [12,13], to attenuate TGFβ-signaling [14] and to be positively involved in apoptosis induction [15,16]. PP2Cβ has also been shown to activate p53 [11], to inhibit MDM2 [11], and to inactivate Cdk2 and Cdk6 [12,13]. Furthermore, it has been shown to activate the proapoptotic protein BAD [20], and to attenuate NF-kB-mediated antiapoptosis [21]. In addition to PP2Cα and PP2Cβ, the recently identified PP2Cs ILKAP and PHLPP have also been implicated in growth regulation; the former has been shown to negatively affect proliferation and malignant transformation [22], and the latter to promote apoptosis and to inhibit tumor growth [29]. Based on these notions, it seems to be reasonable to assume that these four type 2C phosphatases might act as tumor suppressor proteins.

Conversely however, a substantial amount of evidence exists indicating that PP2Cδ (PPM1D/Wip1) is an oncogene; it has been shown to inhibit the activity of four different tumor suppressor proteins (p53, ATM, INK4A and ARF), to inhibit the function of the checkpoint kinases Chk1 and Chk2, to negatively affect base-excision repair and to be overexpressed in various different human malignancies, including e.g. breast carcinomas, ovarian carcinomas, neuroblastomas and medulloblastomas [2,4-6]. These notions exemplify that the properties of PP2Cs cannot be generalized, and they emphasize that in order to be able to attribute clinical significance to the functions of type 2C phosphatases in cell growth, in tumorigenesis and in anticancer therapy, each individual family member needs to be characterized in proper detail.

Here, we have therefore set out to investigate the involvement of the prototypic type 2C phosphatase PP2Cα in growth regulation, in radio- and chemosensitivity, and in tumorigenicity. It was shown that under 'physiological' conditions, the doubling time, the clonogenicity and the membrane integrity were comparable for MCF7-wt and MCF7-si cells (Fig. 1), which indicates that the knockdown of PP2Cα does not affect cell growth in vitro. This notion was confirmed by showing that also upon treatment with different doses of radio- and chemotherapy, the proliferation, the clonogenic survival and the membrane integrity were comparable for wild type and PP2Cα knockdown cells (Figs. 2 and 3).

A large set of cell cycle analyses subsequently demonstrated that the knockdown of PP2Cα did reflect on the cell cycle distribution of the MCF7 cells; a significantly lower percentage (or a significantly shorter residence time) in G0/G1, and a significantly higher percentage (or a significantly longer residence time) in G2/M were found for MCF7-si cells (Fig. 1 and Table 1). These findings are in line with the results published by Chen et al, who have shown that in Drosophila S2 cells, the RNAi-mediated knockdown of CG1906 (i.e. Drosophila PP2C) results in a reduction of the number of cells in G1, and in an increase in the percentages in G2 [36]. In addition, our observations are in line with the data provided by Guillemain et al, who have recently demonstrated that upon the reintroduction of the yeast PP2C gene Ptc2, the number of cells arresting in G2/M after HO endonuclease treatment (which induces DNA double-strand breaks) was significantly lower than that of cells transfected with dysfunctional Ptc2 or with empty vector [37].

Several different mechanisms can be envisioned by which the knockdown of PP2Cα affects the cell cycle. Plausible explanations for the enhanced G1-to-S transition are: I) less PP2Cα is available for dephosphorylating Cdk2 [12,13], which results in an increase in the activity of the Cyclin E – Cdk2 complex, and which thereby promotes entry into the S phase [38-40]. II) Less PP2Cα is available for dephosphorylating Cdk2 [12,13], which results in an increase in the activity of the Cyclin E – Cdk2 complex. Activated Cyclin E – Cdk2 then provides relief from the Rb-mediated repression of the expression of Cyclin A [41,42], thereby enabling Cyclin A to complex with Cdk2 and to induce G1-to-S transition [39,40,43,44]. III) Less PP2Cα is available for dephosphorylating Cdk6 [12,13], which results in an increase in the activity of the Cyclin D – Cdk6 complex. Active Cyclin D – Cdk6 then phosphorylates and inactivates Rb [45-47], thereby enhancing the expression of Cyclin A [41,42] and Cyclin E [48,49], and thereby promoting G1-to-S transition [38-44]. And finally, IV) less PP2Cα is available for inhibiting MDM2 [11]. More MDM2 results in less p53, in less p21, in a reduction of the p21-mediated repression of the activity of the Cyclin E – Cdk2 complex [50,51], and in an induction of the G1-to-S transition [38-40].

The observation that the knockdown of PP2Cα induces the transition from G1 to S was confirmed by evaluating the cell cycle distribution of MCF7-wt and MCF7-si cells upon trypsinization. As most 'normal' cells, MCF7-wt cells presented with substantially increased amounts in G0/G1 at the early time points post trypsinization (i.e. at 6 and 12 h; Table 1). MCF7-si cells, on the other hand, failed to induce a G0/G1 arrest upon trypsinization (Table 1), and also upon chemotherapy, they did not arrest in G0/G1 as effectively as MCF7-wt cells (Fig. 3 and Table 3). Remarkably, however, upon exposure to ionizing radiation, MCF7-si cells did arrest in G0/G1 (Fig. 2E). A potential explanation for this discrepancy is that different signaling pathways are induced by anticancer agents and ionizing radiation in MCF7 cells [52], and that such differences result in a different cell cycle behavior and in an altered induction of cell cycle checkpoints.

Another obvious difference between MCF7-wt and MCF7-si cells was found to be in the number of cells in G2/M; both under basal conditions and upon trypsinization, the percentages of MCF7-si cells in the G2/M phase (or their residence time in G2/M) were significantly higher than those of MCF7-wt cells (Fig. 1 and Table 1). In addition, the induction of the G2/M arrest in response to radio- and chemotherapy was found to be affected by the knockdown PP2Cα. In both cases, it was affected in a time-dependent manner and in a concentration-dependent manner; for radiotherapy, the knockdown of PP2Cα resulted in an earlier and in a stronger induction of the G2 block (Fig. 2 and Table 2), whereas for chemotherapy, it resulted in an earlier and in a less strong induction (Fig. 3 and Table 3). These findings indicate that besides being involved in the G1-to-S transition and the G1 arrest, PP2Cα is also involved in the G2-to-M transition and the G2 arrest.

In the final set of experiments, the tumorigenicity of MCF7-wt and MCF7-si cells was compared. As both types of cells failed to develop tumors under physiological conditions, estrogen-containing hormone pellets and Matrigel were used. The former were implemented because the in vivo growth of MCF7 cells likely depends on the levels of circulating hormones, and the latter was used because it is known to prolong the retention time of (tumor) cells at the site of inoculation. Only upon the combined implementation of the hormone pellets and Matrigel, significant numbers of tumors were obtained for both types of cells; for MCF7-si, tumors grew in 9 out of 10 cases, and for MCF7-wt, tumors developed in 4 out of 8 cases (Fig. 4 and Table 4). In addition to the higher proportion of tumors that developed for MCF7-si, these tumors were also found to be growing more rapidly than their wild type counterparts. These findings were confirmed by showing that in MCF7-si tumors, the expression of the proliferation marker Ki-67 was more abundant and the incorporation of the S-phase marker BrdU was increased (Fig. 4). Our results thus demonstrate that in vivo, MCF7-si cells proliferate more effectively and more rapidly than MCF7-wt cells, and together with the notions that it activates p53 [10,11], inactivates Cdk2 and Cdk6 [12,13], inhibits TGFβ signaling [14] and induces (endothelial cell) apoptosis [15,16], they suggest that PP2Cα may possess tumor-suppressing properties.

To validate this notion, however, additional and more elaborate analyses are necessary, employing e.g. transgenic mice, or cellular constructs in which the expression of PP2Cα can both be induced and reduced conditionally. Histological analyses of human tumor samples, intended to (negatively) correlate the expression of the phosphatase with the grade of malignancy, are also expected to contribute to our understanding of the role of PP2Cα in tumor suppression. And furthermore, PP2Cα-based therapeutic interventions should be evaluated, using either the protein itself, or other means to enhance its expression and/or its activity (e.g. plasmid DNA or low molecular weight agonists). Besides merely increasing our knowledge on the involvement of this type 2C phosphatase in inhibiting tumor growth, such analyses may also give rise to new therapeutic entities for treating advanced solid malignancies, and they likely also lead to new insights on other physiological functions of PP2Cα.

## Conclusion

In summary, we here show that the siRNA-mediated knockdown of PP2Cα does not affect the in vitro proliferation, the clonogenic survival, the membrane integrity and the radio- and chemosensitivity of MCF7 cells. It does, however, substantially reflect on their cell cycle distribution: for MCF7-si cells, the number of cells in G0/G1 was reduced, the induction of the G1 block was attenuated, the number of cells in G2/M was increased and the induction of the G2 block was enhanced. In addition, the knockdown of PP2Cα affected the tumorigenic potential and the in vivo proliferation of the MCF7 cells, which were both found to be significantly higher for cells expressing attenuated amounts of the protein. Based on these findings, we conclude that PP2Cα plays a role in the regulation of the cell cycle, in the induction of cell cycle checkpoints and in tumor suppression.

## Methods

### Plasmid preparation

The pSUPER-Retro vector [31] was prepared for ligation by double-cutting with HindIII and BglII enzymes, and by purifying the linearized vector using agarose gels. Lyophilized synthetic 64-basepair long DNA oligonucleotides were dissolved in 50 μl of ultra-pure water. The oligonucleotide sequences used were: I) PP2Cα-siRNA-forward: 5'-GATCCCCCACATGAGAGTTATGTCAGTTCAAG AGACTGACATAACTCTCATGTGTTTTTGGAAA-3' (the siRNA target sequence from the PP2Cα gene is underlined), and II) PP2Cα-siRNA-reverse: 5'-AGCT TTTCCAAAAACACATGAGAGTTATGTCAGTCTCTTGAACTGACATAACTCTCATGTGGGG-3'. One microliter of each oligonucleotide was annealed in 48 μl of annealing buffer (4 min incubation at 95°C, 10 min at 70°C and slow cooling to 4°C). Two microliters of the annealed oligos were then phosphorylated at their 5'-end using 10 U of T4 polynucleotide kinase (PNK; New England Biolabs). Before ligation, the molar concentration of the purified vector and of the phosphorylated oligos were determined. The ligation was performed in a volume of 20 μl for 2 h at room temperature, according to the manufacturer's instructions, i.e. using 3 U of T4 DNA ligase (Promega) and a 10–20-fold molar excess of the oligos. The ligation product was directly transformed into competent cells. The presence of the insert in growing colonies was confirmed using plasmid mini-preparation and double-cutting with EcoRI and HindIII enzymes.

### Cell transfection and tissue culture

Wild type MCF7 (human breast adenocarcinoma) cells were obtained from ATCC. PP2Cα siRNA-expressing MCF7 cells were prepared by growing wild type MCF7 cells to 50–70 % confluency, and by then transfecting them with the abovementioned oligonucleotide-containing plasmid using Lipofectamine (Invitrogen). It should be noted here that besides the polymerase-III H1-RNA gene promoter and the genetic insert encoding for PP2Cα-siRNA, the pSUPER-Retro vector also contains a puromycin resistance gene, in order to allow for the selection of positively transfected clones [31]. After 6 h of incubation at 37°C, the transfection mixture was removed and it was replaced with complete DMEM (Gibco), i.e. supplemented with 1% glutamine (Gibco) and 10% FCS (Gibco). The siRNA-expressing cells were subsequently grown for an additional 12 h, and from then on, the concentrations of puromycin (Sigma-Aldrich) were gradually increased from 1 to 20 μg/ml. Several positively transfected clones were obtained, and these were cultivated in complete DMEM containing 20 μg/ml of the selection agent.

### Proliferation and viability assays

The doubling time of wild type and PP2Cα siRNA-expressing MCF7 cells was determined by seeding 1 * 10^5 ^cells into 6-well plates (3 wells per time point). At several time points after seeding, the cells were trypsinized and harvested, and the number of cells in each well was quantified using an automated cell counter (Coulter Counter ZM). The proliferation of the cells upon treatment with radio- and chemotherapy was determined by means of the sulphorhodamine B (SRB) assay [32]; hereto, 1 * 10^5 ^cells were seeded into 6-well plates and they were grown for 5–8 days. Then the cells were fixed, stained and dried, the SRB dye was extracted, it was transferred to a 96-well plate, and the absorbance of each of the wells was analyzed spectrophotometrically at 540 nm. The clonogenic survival of the cells was determined by means of the colony formation assay (CFA); 2000 cells were seeded into 6-well plates and they were grown for 10–14 days. Then, they were fixed and stained with crystal violet, and the number of colonies (i.e. spatially separated plagues consisting of at least 50 cells) was counted. The membrane integrity of the cells was investigated by quantifying the uptake of propidium iodide (PI; a membrane-impermeable dye that only enters and stains membrane-damaged and dead cells). 1 * 10^6 ^Cells were seeded into T75 culture flasks, and at several time points post treatment, they were trypsinized, harvested and incubated with PI. The uptake of PI was analyzed on a FACS Calibur (Becton Dickinson).

### Cell cycle analysis

The cell cycle distribution of wild type and PP2Cα siRNA-expressing MCF7 cells was determined by seeding 1 * 10^6 ^cells into T75 culture flasks. At several predefined time points after seeding (and after radio- or chemotherapy), the cells were trypsined, harvested, resuspended in 1 ml of -20°C methanol and stored until analysis. Immediately prior to analysis, the cells were washed with culture medium and resuspended in 1 ml of PBS containing 0.1% of NaN_3_. Then, 50 μg of PI were added to each vial and the samples were analyzed on a FACS Calibur (Becton Dickinson).

### Radio- and chemotherapy

Radiotherapy was applied by exposing the cells (in well-plates or in culture flasks) to different doses of ^60^Cobalt γ-radiation. Radiotherapy was delivered by means of the Siemens Gammatron S, at a dose rate of 0.5 Gy/min. Doxorubicin was obtained from Sigma-Aldrich. Gemcitabine was kindly provided by Eli Lilly. Both agents were of appropriate analytical grade (>99.8%).

### In vivo analysis

All experiments involving animals were approved by an external committee for animal welfare and were performed according to international and institutional guidelines. The tumorigenicity of wild type and PP2Cα knockdown MCF7 cells was investigated by inoculating 1 * 10^7 ^cells into both hind limbs of immunodeficient nude mice. As both types of cells failed to develop tumors under 'standard' conditions, their tumorigenicity was also evaluated upon the implementation of estrogen-containing hormone pellets (0.36 mg 17β-Estradiol per pellet; Innovative Research of America) and Matrigel (Matrigel Basement Membrane Matrix; Becton Dickinson). The tumors that developed upon the combined implementation of hormone pellets and Matrigel were harvested at day 37 after inoculation. Both for MCF7-wt and for MCF7-si, three animals that had developed tumors were injected with BrdU 24 h before harvesting, and the incorporation of the proliferation marker was analyzed by means of FACS analysis.

### Immunohistochemistry

Cryosections (6 μm) were prepared using the Leica Frigocut 2800 E and they were fixed in methanol/acetone. Prior to staining, the cryosections were blocked with the Image-iT FX signal-enhancer (Molecular Probes) and/or with 5% human serum albumin (HSA; Sigma-Aldrich). Then, they were incubated for 1 h with a 1/100 dilution of rabbit anti-PP2Cα (3S1; Prepared at Tel Aviv University [10,11]), or with a 1/50 dilution of rabbit anti-Ki-67 (Proliferation marker; sc-15402; Santa Cruz). Upon three PBS washes, the sections were incubated with a 1/100 dilution of donkey anti-rabbit Alexa-Fluor-488 (A21206; Molecular Probes), or with a 1/250 dilution of goat anti-rabbit-HRP (P0448; DakoCytomation). Nuclear counterstaining was performed using DAPI or Haematoxilyn. 3-Amino-9-ethylcarbazole (AEC) was used as an HRP-responsive dye for light microscopy.

### Statistical analysis

All values are expressed as average ± standard deviation. The two-tailed Student's t-test was used to assess if the differences between the various experimental groups were significant. P < 0.05 was considered to represent statistical significance.

## Competing interests

The author(s) declare that they have no competing interests.

## Authors' contributions

TL, JD, SL and PH designed the experiments, BS and SL prepared the knockdown cells, TL, PP and VE conducted the viability and the cell cycle experiments, TL and PP performed the in vivo and the immunohistochemical analyses, and TL, PP, JD and PH prepared the manuscript.
